# Variability and directionality of inferior olive neuron dendrites revealed by detailed 3D characterization of an extensive morphological library

**DOI:** 10.1007/s00429-019-01859-z

**Published:** 2019-03-30

**Authors:** Nora Vrieler, Sebastian Loyola, Yasmin Yarden-Rabinowitz, Jesse Hoogendorp, Nikolay Medvedev, Tycho M. Hoogland, Chris I. De Zeeuw, Erik De Schutter, Yosef Yarom, Mario Negrello, Ben Torben-Nielsen, Marylka Yoe Uusisaari

**Affiliations:** 1 Department of Neurobiology, Institute of Life Sciences and Edmond and Lily Safra Center for Brain Sciences, Hebrew University, Jerusalem, Israel; 2 Netherlands Institute for Neuroscience, Royal Netherlands Academy of Arts and Sciences, Amsterdam, The Netherlands; 3 Department of Neuroscience, Erasmus MC, Rotterdam, The Netherlands; 4 Computational Neuroscience Unit, Okinawa Institute of Science and Technology Graduate University, Onna, Okinawa 904-0495 Japan; 5 Neurolinx Research Institute, La Jolla, CA USA; 6 Neuronal Rhythms in Movement Unit, Okinawa Institute of Science and Technology Graduate University, Onna, Okinawa 904-0495 Japan

**Keywords:** Dendritic morphometry, Sparse viral labeling, Network structure, Brainstem, Olivo-cerebellar system, Neuron reconstructions

## Abstract

The inferior olive (IO) is an evolutionarily conserved brain stem structure and its output activity plays a major role in the cerebellar computation necessary for controlling the temporal accuracy of motor behavior. The precise timing and synchronization of IO network activity has been attributed to the dendro-dendritic gap junctions mediating electrical coupling within the IO nucleus. Thus, the dendritic morphology and spatial arrangement of IO neurons governs how synchronized activity emerges in this nucleus. To date, IO neuron structural properties have been characterized in few studies and with small numbers of neurons; these investigations have described IO neurons as belonging to two morphologically distinct types, “curly” and “straight”. In this work we collect a large number of individual IO neuron morphologies visualized using different labeling techniques and present a thorough examination of their morphological properties and spatial arrangement within the olivary neuropil. Our results show that the extensive heterogeneity in IO neuron dendritic morphologies occupies a continuous range between the classically described “curly” and “straight” types, and that this continuum is well represented by a relatively simple measure of “straightness”. Furthermore, we find that IO neuron dendritic trees are often directionally oriented. Combined with an examination of cell body density distributions and dendritic orientation of adjacent IO neurons, our results suggest that the IO network may be organized into groups of densely coupled neurons interspersed with areas of weaker coupling.

**Electronic supplementary material:**

The online version of this article (10.1007/s00429-019-01859-z) contains supplementary material, which is available to authorized users.

## Introduction

The inferior olive (IO) provides the sole source of climbing fibers that evoke potent complex spikes in cerebellar Purkinje neurons (PNs), and thereby plays a critical role in controlling cerebellar function (Azizi 2007; Jacobson et al. 2008; Ito 2013; Llinás 2014; Ten Brinke et al. 2018; Streng et al. 2018). The neurons within the IO are exclusively interconnected by dendro-dendritic gap-junctions (GJs; Sotelo et al. 1974; De Zeeuw et al. 1989; Placantonakis et al. 2004). The GJ-mediated signaling shapes subthreshold oscillations (STOs) and spike timing among coupled IO neurons (De Zeeuw et al. 1998, 2003; Long et al. 2002; Blenkinsop and Lang 2006; Jacobson et al. 2009; Kitazawa and Wolpert 2005; Welsh et al. 1995; Lampl and Yarom 1997; Loewenstein et al. 2001; Manor et al. 1997; Placantonakis et al. 2006; Torben-Nielsen et al. 2012). Thus, the dendritic layout which determines connectivity within the nucleus is at the core of the spatiotemporal patterning of IO network activity.

Morphologically, IO neurons have historically been classified into “curly” and “straight” types (Ramón y Cajal 1995; Scheibel and Scheibel 1955; Foster and Peterson 1986). The “curly” type is characterized by complex curled dendritic trees that branch and bend profusely within a very small volume of the neuropil around the soma. In contrast, the “straight” neurons have dendrites sparsely occupying a much larger volume. As GJs are overwhelmingly located on the IO neuron’s dendrites, the different dendritic shapes must lead to different connectivity profiles. Nevertheless, relatively little is known about the structural properties of IO neurons, and quantitative descriptions of different IO neuron morphologies are lacking. One reason for this is that anatomical investigations have long been limited to the examination of two-dimensional projections of neuronal structures. Using more advanced labeling techniques and detailed confocal imaging, we can now fully reconstruct and accurately quantify complex dendritic morphologies in 3D.

In this work we constructed an extensive library of IO neuron morphologies and give a detailed quantitative description of the variability in their morphological properties and the spatial arrangement of their dendritic arbors. Our results reveal that dendritic tree shapes span a continuum between the classically described “curly” and “straight” IO neuron morphologies and that dendritic trees are often directional. These findings have important implications for our understanding of connectivity in the IO network.

## Methods

All animal experimental procedures were approved by the Hebrew University’s Animal Care and Use Committee, and the animal experiment committee of the Royal Netherlands Academy of Arts and Sciences (DEC-KNAW) which follows the European guidelines for the care and use of laboratory animals (Council Directive 86/6009/EEC).

### Single neuron labeling

Sparse viral labeling of neurons was achieved by injecting a cre-dependent fluorophore-expressing virus mixed with a highly diluted cre-expression virus into the IO of juvenile or adult mice (6 weeks to 4 months old; all animals were at least 10 weeks old after the viral transfection period). The Cre-expression virus (AAV9.CamKIIa.cre, Penn Vector Core) was diluted (1:3000, 1:3500 or 1:4000) with saline in multiple steps, taking care to mix well at each step. The diluted viral suspension was then mixed 1:1 with a loxed GFP-expression virus (AAV9.CAG.flex.eGFP.bGH, Penn Vector Core). Mice were anaesthetized using a mixture of ketamine and xylazine (100 mg/kg and 20 mg/kg) and head-fixed into a stereotaxic device. The skull over the IO was exposed through a single incision into the skin and scraping away some of the soft tissue covering the area. A single craniotomy, ~ 2 mm wide was then drilled in the skull, centered around the midline just behind the posterior suture. ~500 nL of the mixture of *Cre* and *lox* viruses was then slowly injected at 6.5 mm depth, bilaterally to the midline using air pressure. After 4–6 weeks incubation time, mice were deeply anesthetized with pentobarbital and fixed through transcardiac perfusion with 4% paraformaldehyde (PFA) in phosphate-buffered saline (PBS), and brains were post-fixed overnight in the same solution. The brains were then washed in PBS and the brain stem cut into 150 µm-thick sections in coronal or sagittal plane using a Leica VT1000S or Leica VT1200S vibratome (Leica Biosystems, Germany) and subsequently mounted with prolong gold antifade mounting medium (RI 1.47; Thermo Fisher Scientific, MA) under #1.5 coverslip glass (Thermo Fisher).

Dye-filling of IO neurons was achieved during in vitro patch-clamp experiments on acute brainstem slices (performed by N.V. or S.L., for the purpose of other projects). Alexa-labeling of IO neurons was done in 200 µm-thick coronal brainstem slices prepared following the “hot” procedure (Huang and Uusisaari 2013; Ankri et al. 2014); in brief, adult mice (3–12 months old) of either sex were deeply anesthetized with pentobarbital, decapitated and their brain stem extracted from the skull while continuously kept in oxygenated artificial cerebrospinal fluid (ACSF) warmed to a temperature of 30–35 °C. The ACSF was composed of (in mM) 126 NaCl, 3 KCl, 1.2 kH_2_PO_4_, 26 NaHCO_3_, 10 glucose, 2.4 CaCl_2_, 1.3 MgSO_4_ and continuously bubbled with carbogen (95% O_2_/5% CO_2_). Slices were then incubated at 35 °C for at least half an hour and then at room temperature. Fluorescent labeling of IO neurons was achieved by adding 20–50 µM Alexa-594 or Alexa-488 Hydrazide (Thermo Fisher Scientific, MA) to a patch pipette solution containing (in mM) 4 NaCl, 140 K-gluconate, 10 HEPES, 0.01 EGTA, 0.001 CaCl_2_ and 4 Mg-ATP (pH adjusted with KOH to 7.2–7.3, osmolality 290–310 mOsm) during whole-cell recordings performed at room temperature. Whole-cell configuration was maintained for at least half an hour and slices were incubated for an additional half hour after recordings were terminated to allow dye to spread through dendrites. Slices were subsequently preserved by fixation in 0.1 M PBS containing 1% PFA for 30 min and then washed and stored in PBS until mounted with Vectashield (RI 1.45; Vector laboratories, CA) and coverslipped. The biocytin-labeling experiments differed on several points: juvenile (4–8-week-old) mice of either sex were anesthetized with isoflurane, and their brain stem extracted and sliced in ice-cold ACSF. Slices were cut sagittally and then incubated at 35 °C for half an hour and at room temperature for at least half an hour, before being transferred into a recording chamber maintained at ~ 32 °C. The ACSF had the same composition as used in the Alexa-labeling experiments, as was the patch pipette solution except in that it contained 5 EGTA and 0.5 CaCl_2_, and 0.1–0.5% (w/v) biocytin (Sigma) was added. After recordings were completed, slices containing biocytin-filled neurons were fixed in 0.1 M PBS containing 4% PFA overnight at 4 °C. Slices were then washed three times (0.1 M PBS, 10 min at 4 °C), incubated with Alexa Fluor 594-conjugated streptavidin (Life technologies, 2 mg/ml) and 0.6% Triton X-100 (Sigma) in 0.1 M PBS (4 h at 4 °C), washed three times (0.1 M PBS, 10 min at 4 °C), mounted with Dako glycergel fluorescence mounting medium (RI 1.47–1.50; Dako) and coverslipped.

In our examinations of hundreds of IO neurons in both sagittal and coronal brain stem slices we noted no overt differences in the morphologies’ orientations relative to the confocal *z*-axis, or any tendency for “curlier” or “straighter” neurons to be more prevalent in juvenile or adult mice; regardless of the experimental conditions, labeled morphologies exhibited extensive heterogeneity covering the full range from “curly” to “straight”. We therefore chose to consider all the available material together and select only the most complete morphologies (see below) for inclusion in our library.

### Recovery and reconstruction of morphologies

The labelled material was examined and imaged using confocal microscopy (Leica SP5 and SP8, Leica Microsystems, Germany; Zeiss LSM 510, 710, 780 and 880, Zeiss, Germany). Each mounted section was first scanned with low magnification (10×) and a maximal projection of the slice was created to record the position of the neurons within the IO volume and select candidates for high-resolution stack acquisition.

High-magnification confocal image stacks were obtained with either 40 or 63× plan-Apochromat objectives (NA 1.25–1.3) as were available at each confocal system, so that resolution ranged from 0.11 to 0.38 µm/pixel in *XY* plane. The sections were oversampled in *z*-dimension (ranging 0.1–0.3 µm/*z*-step) to support correction of the *z*-axis values due to shrinkage factor. The morphologies were manually reconstructed using the Vaa3D software (Peng et al. 2010), taking care that the reconstructions end up as sorted trees with a single root. The shrinkage was estimated from the thickness of the mounted section (as measured by confocal visualization) relative to the fresh section and the final reconstructions were expanded in *z*-dimension to account for the shrinkage (ranging 1.5–3×).

To ensure that the overall dendritic shape of the morphologies in our library was not distorted, morphologies were carefully selected for inclusion based on the completeness of their 3D reconstruction. Morphologies that appeared skewed, due to optical or physical distortions, were discarded from analysis. Distal and/or very thin dendrites were occasionally difficult to reconstruct in entirety due to decreasing signal/noise ratio, and reconstructions were discarded if multiple disconnected fine branches could be observed around a reconstruction’s dendrite tips in the confocal image stack. We also kept track of the number dendrite tips occurring at the slice surface counting these as “cut tips” and discarded any morphologies that had more than half of tips cut, or that had one or more proximal dendrites cut at < 50 µm path length. Out of the hundreds of neurons examined in confocal image stacks, ~ 150 morphologies were reconstructed, and a total of 36, 27 and 29 morphologies were selected for the viral, Alexa- and biocytin-labeled datasets, respectively. The selected morphologies will be submitted to NeuroMorpho.org.

### Quantification of morphological properties

The included morphologies were first inspected by the authors and subjectively labeled as being either “straight”, “curly” or “ambiguous” (18, 44 and 30 out of 92 morphologies, respectively). Subsequently, the 25 parameters, covering both “within-tree” and “whole-tree” variables (Uylings and van Pelt 2002) were obtained as extracted by Vaa3D or by custom scripts in MatLab and btmorph (Torben-Nielsen 2014). The complete list of measurements, together with their definition, is provided in Table 1.

**Table 1:** The morphometric measures used in the study

Measure #	Name	Description
1	Number of stems	Number of primary dendrites
2	Stem diameter—mean	Average diameter of dendrite stems (µm)
3	Stem diameter—sum	Sum of the diameter of all dendrite stems (µm)
4	Stem diameter—maximum	Maximal diameter of dendrite stems (µm)
5	Stem directionality	Directionality of dendrite stems; for definition see “Methods”
6	Dendrites—total length	The summed length of all of a neuron’s dendrites (µm)
7	Dendrite diameter—mean	Average diameter of the dendrites (µm)
8	Dendrites—longest single path length	Longest soma-to-tip dendrite path length (µm)
9	Number of bifurcations	Number of bifurcation points on the dendritic tree
10	Local bifurcation angle—mean	Angle formed at the vertex of a bifurcation, averaged over all bifurcations
11	Remote bifurcation angle—mean	Angle to the tips of two daughter branches of a bifurcation, averaged over all bifurcations
12	Number of branches	Number of segments (between two branch points or between a branch point and a tip)
13	Branch order—maximum	The number of branches coming off the most-branching dendrite on the tree
14	Number of tips	Number of dendrite terminal points within the imaged slice
15	Number of cut tips	Number of dendrites running out of the imaged slice
16	Number of tips—total	Total number of dendrite terminal points on the reconstructed morphology
17	Soma area	Area of the 2D projection of the soma
18	Hull volume	Volume of the convex hull containing all of the neuron’s dendrites (µm^3^)
19	Soma-to-hull distance	Smallest distance between the soma and the hull containing all of the neuron’s dendrites (µm)
20	Soma-to-center of gravity distance	Distance between the soma and the average of all points of the reconstructed morphology (µm)
21	Reach—maximum	Furthest reach of the dendritic tree away from the soma (µm)
22	Straightness	Maximal reach divided by maximal path length
23	Mean contraction	Furthest reach divided by longest path length of each tree arising from the primary dendrites, averaged over the number of primary dendrites
24	Hausdorff dimension	Measure of fractal dimension (Mizrahi et al. 2000)
25	Mean fragmentation	Number of compartments that form a branch between two bifurcation points, or between a bifurcation and a terminal tip

Numbers in the first column are used to reference to the measures in Fig. 3c. “Compartment” refers to the variable-length nodes of reconstruction within which the dendrite thickness and shape is uniform

Three of the measures obtained by custom scripts were defined as follows. Soma-border distance was defined as the shortest distance of the reconstruction root node to the extrapolated convex hull of the full reconstruction. Soma-center of mass distance was defined as the distance of the root node to the average location of all the nodes of the reconstruction. Stem directionality was defined as the length of the vector obtained by sum of all vectors formed from reconstruction root node to the first nodes of each dendrite, normalized by the number of stem vectors; in this way, a soma with one dendrite would have a directionality value of 1, whereas a soma with dendrites stemming evenly around the cell body would approach directionality of 0.

### Statistical analyses

Statistical analyses were performed in R (R Core Team 2018; Wickam et al. 2017, 2018; Fox and Weisberg 2011; Wickam 2016; Revelle 2018; Ogle 2018; Peterson and Carl 2018; Kassambara and Mundt 2017; Venables and Ripley 2002), unless stated otherwise. The assumption that data are sampled from a normal distribution was rejected for almost all measures based on the Shapiro–Wilk normality test (*p* < 0.1 in each data set for all measures except number of stems and average local bifurcation angle). Therefore, correlation estimates and *p* values were calculated using Spearman’s rank correlation test, and group-level comparisons in mean and variance were calculated using Welch’s ANOVA and Levene’s test for equality of variance, respectively.

For performing PCA on the morphometric data per dataset, the values of all morphometric features were scaled and centered to have zero mean and unit variance. We then applied *K*-means clustering into two clusters to the data as represented along the first three components of the decomposition (which resulted in nearly identical clustering as applying *K*-means clustering to the data represented along the first two PCs only).

On individual morphologies, PCA was performed in MatLab and applied to the *x*-, *y*-, *z*-coordinates of each point on the reconstruction without re-scaling the data since variance has the same units in each dimension of 3D space.

### Reconstruction and analysis of IO soma distribution

For analyzing the spatial distribution and clustering of IO somata, we used two mice obtained from a PDX-cre (Song et al. 2010) X Ai9 (Madisen et al. 2010) mating, resulting in strong expression of tdTomato in IO neurons. The mice were perfusion-fixed and their brains sectioned and mounted as described above, and all somata on one hemisphere in both animals were reconstructed manually for each subnucleus using Fiji software (Schindelin et al. 2012).

The density distribution of IO somata was estimated by 3D binning the somata in voxels of 10 µm^3^, and subsequently applying an isotropic 3D-gaussian kernel to account for binning artifacts. The standard deviation parameter utilized for the 3D kernel was 4. To test the null-hypothesis that the density of somata was isotropic, the distribution of voxel densities of the data was compared to a volume bootstrapping the somata densities assuming a uniform density. Thus, the bootstrap was constructed by drawing somata counts from a uniform distribution within the bounded volume formed by the non-zero somata voxels. The density per voxel in the uniform distribution is simply the total somata count in the actual subnucleus divided by the total volume included in non-zero voxels. A two-sample Kolmogorov–Smirnov test was used to compare the distributions.

Presence of local soma clusters was examined using the DBSCAN algorithm (Ram et al. 2010) implemented in MatLab. This algorithm assigns cluster membership to any group of at least *N* somata where any one soma within the cluster is at most *D* µm removed from another cluster member. Clustering was explored for values of *D* ranging from 15 to 100 µm and values of *N* ranging from 3 to 20.

## Results

### Variability of IO dendritic morphology

**Fig. 1:**
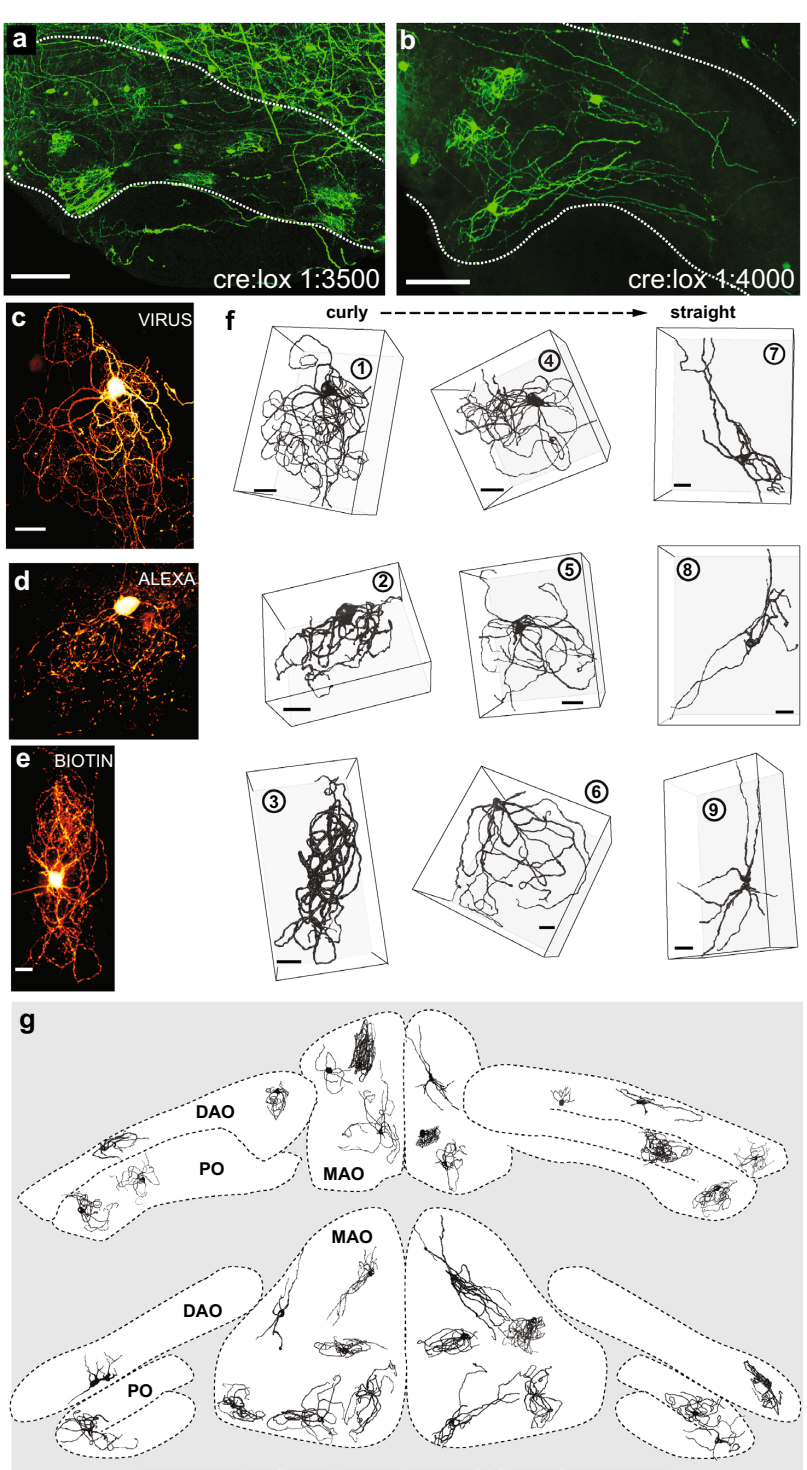
Fluorescent labeling of IO neurons reveals complex morphologies. **a** Maximal projection of a 100 µm-thick confocal image stack of a coronal brain stem slice labeled sparsely by viral transfection (1:3500 dilution of cre-expression virus). IO borders are marked with a white dotted line. Scale bar 100 µm. **b** as in **a**, but with 1:4000 dilution. **c–e** Confocal image *z*-stacks exemplifying “very curly” IO neurons as revealed by viral (**c**), Alexa-594 (**d**) or biocytin (**e**) staining. Scale bar 20 µm. **f** Examples of reconstructed morphologies from the three data sets (as in **c–e**) ranging from “very curly” (left column, same examples as shown in **c–e**) to “straight” (rightmost column). Note that the scale varies between subpanels and perspective; scale bars represent 20 µm in the *xy* plane. Encircled numbers denote reconstruction IDs as referred to in the text. **g** A composite drawing showing the shape and orientation of a selection of the morphologies within the volume of the IO. Note the presence of curly and straight neurons in all subnuclei (abbreviations: *PO* principal olive, *DAO* dorsal accessory olive, *MAO* medial accessory olive)

We used a sparse viral labeling technique to induce strong fluorescent labeling in a small number of neurons in a given IO (Fig. 1a, b) as well as IO neurons that were labeled using either Alexa (488 or 594) or Biocytin during in vitro patch-clamp experiments (see “Methods”). Confocal image stacks were acquired from the labeled tissue and a total of 90 manually reconstructed morphologies were analyzed (see “Methods”). Except for two neurons from the dorsal cap of Kooy (DCK)-subnucleus (which has been shown to be phenotypically distinct from the main IO subnuclei; Urbano et al. 2006), neurons from all IO subnuclei were included in our analyses. The different methods of neuronal labeling lead to some variability in the quality of the confocal image stacks; specifically, the viral-labeled material was of higher quality regarding the ratio between signal strength and background noise. Nonetheless, qualitative differences between morphologies reconstructed from the differently labeled materials were not immediately apparent (see Fig. 1c–f). To exemplify morphologies from each of the three data sets, Fig. 1c–e show maximal *Z*-projections of confocal stacks from viral-, Alexa- and biocytin-labeled data, respectively; the corresponding reconstructed morphologies are shown in the left column in Fig. 1f. Additional examples of morphologies reconstructed from the three datasets are shown in the middle and right columns of Fig. 1f, indicating a progression from “very curly” (left column) to “very straight” (right column) morphologies in each of the three data sets.

Examining the full morphological library, both “clearly curly” (30 out of 90; Fig. 1f, left column) and “clearly straight” morphologies (16 out of 90; Fig. 1f, right column) could be subjectively identified. However, categorical distinction was ambiguous, as a significant portion of the morphologies could not be easily classified (44 out of 90; examples are shown in Fig. 1f, middle column).

It has been previously considered that IO neurons with subjectively straight and curly appearance would be anatomically segregated into different parts of the olivary nucleus (Scheibel and Scheibel 1955; Ruigrok et al. 1990). However, we found that “curly” and “straight” neurons could be found within each of the main IO subnuclei. This is demonstrated in Fig. 1g, where morphologies from different sources are shown at their anatomical locations approximated at two different levels of the anterio-posterior axis (see “Methods”). These results demonstrate extensive morphological heterogeneity in IO neuron dendritic morphologies across all subdivisions of the nucleus.

### Quantitative analyses reveal a continuum in neuronal morphology

**Fig. 2:**
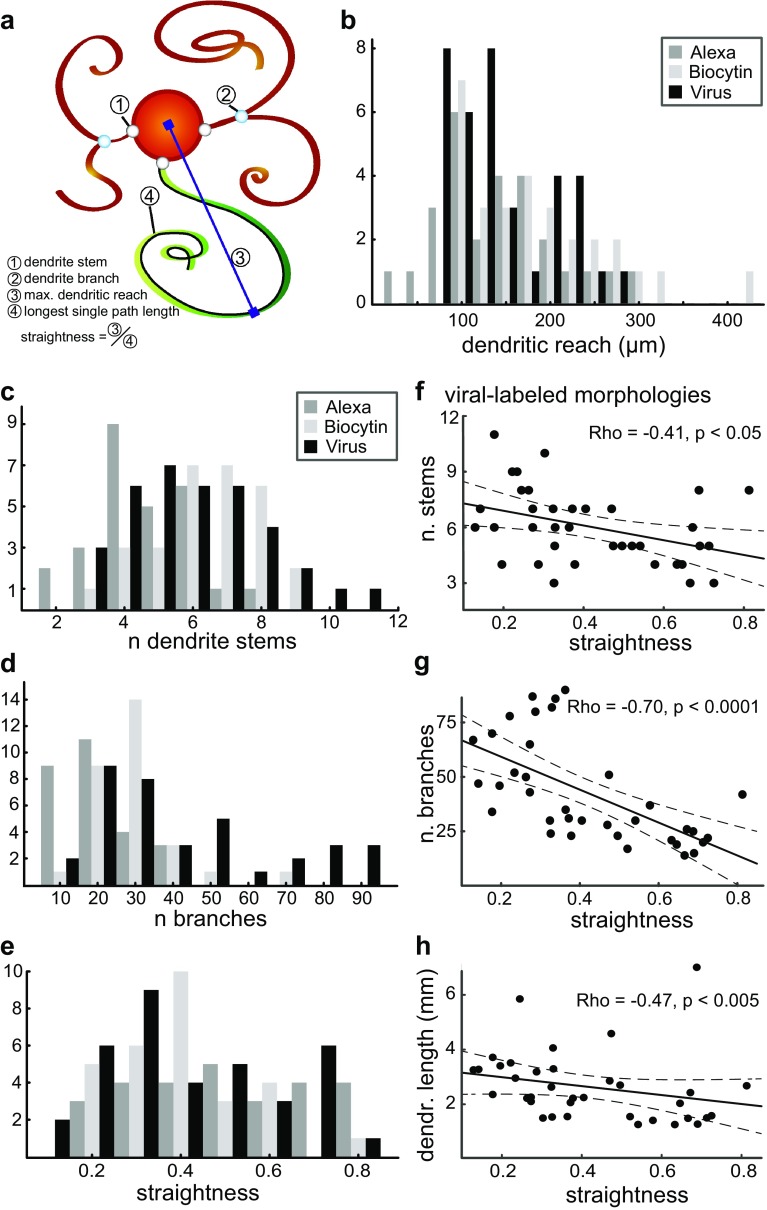
Morphological properties of IO neurons quantified. **a** Schematic illustration of some of the basic morphological parameters used to characterize the dendritic morphologies. Maximal dendritic reach is defined as the furthest reach of the dendritic tree away from the soma; the longest single path length is defined as the longest soma-to-tip path length on a dendritic tree; and straightness is defined as the maximal dendritic reach divided by the longest single dendrite path length. For a list of all morphometric parameters and their definitions, see Table 1. Distributions of maximal dendritic reach (**b**), number of dendrite stems emerging from the soma (**c**), number of branch points on the dendritic trees (**d**) and straightness (**e**) in each of the three data sets; shadings refer to morphologies recovered using different labeling methods as indicated. Distributions of number of dendrite stems (**f**), number of branch points (**g**) and total dendritic length (**h**) with respect to straightness in the viral-labeled data; the same distributions in the Alexa- and biotin-labeled data sets are displayed in Supplementary Fig. 1. Reported correlation statistics represent the strength and direction (Rho) and significance level (*p*) calculated using Spearman’s rank correlation test (see “Methods”). Correlations between straightness and all other morphological measures are reported in the right half of Table 2 for each of the three data sets

While the ambiguity of dendritic morphologies seemed to rule out clear classification, we investigated whether features distinguishing between IO cell classes could be revealed using a quantitative approach. To this end we measured 25 morphometric parameters from each of the reconstructed neurons (see Table 1 and “Methods” for measurement definitions). The measured parameters included basic ones such as the number of dendrite stems, number of branches, dendritic path length and maximal reach (see Fig. 2a). We also measured a number of parameters aimed at describing the overall shape of the dendritic trees; most prominent among these (as explained below) is “straightness”, which was defined as maximal reach divided by the longest single dendrite path length.

**Table 2:** Significance of group-level differences in mean (left column) and variance (right column)

Measure names	Welch’s ANOVA	Levene’s test for equality of variance
Number of stems	< **0.0001**	0.2562
Stem diameter—mean	**0.0129**	0.7094
Stem diameter—sum	< **0.0001**	**0.0071**
Stem diameter—maximum	**0.0004**	0.4644
Stem directionality	0.5522	0.8356
Dendrites—total length	< 0.0001	0.0743
Dendrite diameter—mean	**0.0005**	0.6540
Dendrites—longest single path length	< **0.0001**	0.3177
Number of bifurcations	< **0.0001**	< **0.0001**
Local bifurcation angle—mean	0.2153	0.1322
Remote bifurcation angle—mean	0.7173	0.1410
Number of branches	< **0.0001**	< **0.0001**
Branch order—maximum	**0.0002**	**0.0295**
Number of tips	**0.0065**	**0.0460**
Number of cut tips	**0.0006**	0.1199
Number of tips—total	< **0.0001**	**0.0332**
Soma area	< **0.0001**	**0.0279**
Hull volume	**0.0152**	0.3412
Soma-to-hull distance	**0.0076**	0.0862
Soma-to-center of gravity distance	**0.0138**	0.2072
Reach—maximum	0.0522	0.3715
Straightness	0.0543	0.0755
Mean contraction	< **0.0001**	0.2088
Hausdorff dimension	**0.0002**	**0.0123**
Mean fragmentation	**0.0016**	0.3353

Values reflect *p* levels calculated using Welch’s ANOVA (left) and Levene’s test (right), for each of the morphological parameters as indicated in each row. Values highlighted in bold indicate measures on which morphological parameter distributions across the three data sets were significantly different in their mean/variance, respectively

Examining the distributions of morphological parameter values, we noted that there are some differences between the three groups of neurons reconstructed from material obtained using different labeling methods. The outcome of statistical tests performed to assess differences across groups are summarized in Table 2 and show that group means were significantly different on almost all measures. Thus, we performed quantitative analyses of morphometric measures on each of the three data sets separately.

In an ideal and simple case, a distinction between groups is implied by a clear bi- or multimodal distribution in one or more features. However, we observed no immediately apparent groupings in the distributions of any of the measured parameters. To exemplify the variability in morphological parameter distributions, histograms of maximal reach, number of stems, number of branches, total dendrite length and straightness are shown in Fig. 2b–e for each data set as indicated in the legend, demonstrating that there are no clear multimodal distributions in any of the data sets. Nonetheless, it was evident that IO neurons do not form a single population with normally distributed inter-individual variability: as shown in Table 3, we found that in each data set, the null-hypothesis that data are sampled from a single normal distribution should be rejected for almost all measures except number of stems and local bifurcation angle. To enumerate the distributions of the measured morphological parameters, Table 4 displays the minimal, mean, median and maximal values of each parameter distribution in each data set. Taken together, while we should conclude that the observed morphological heterogeneity is unlikely to result from normally distributed inter-individual variability in a single neuronal population, we did not find any single morphometric parameter that would clearly distinguish between morphologically different classes of neurons.

**Table 3:** Likelihoods of normality

Measure names	Viral-labeled	Alexa-labeled	Biotin-labeled
Number of stems	0.1355	0.1881	0.1440
Stem diameter—mean	0.1712	**0.0078**	**0.0366**
Stem diameter—sum	0.8794	0.5099	**0.0007**
Stem diameter—maximum	**0.0068**	**0.0097**	**0.0298**
Stem directionality	0.1491	*0.0814*	0.6311
Dendrites—total length	**0.0003**	*0.0689*	0.1586
Dendrite diameter—mean	0.2036	0.6204	**0.0056**
Dendrites—longest single path length	**0.0010**	0.2313	**0.0009**
Number of bifurcations	**0.0012**	0.1020	**0.0007**
Local bifurcation angle—mean	0.2128	0.7908	0.5166
Remote bifurcation angle—mean	0.2437	*0.0913*	0.7735
Number of branches	**0.0015**	0.1663	**0.0010**
Branch order—maximum	*0.0812*	0.0066	**0.0052**
Number of tips	*0.0892*	0.0368	**0.0040**
Number of cut tips	*0.0826*	0.2140	**0.0004**
Number of tips—total	**0.0144**	0.1568	**0.0003**
Soma area	0.8722	**0.0006**	**0.0408**
Hull volume	< **0.0001**	**0.0059**	< **0.0001**
Soma-to-hull distance	**0.0043**	**0.0039**	**0.0011**
Soma-to-center of gravity distance	< **0.0001**	< **0.0001**	*0.0613*
Reach—maximum	**0.0019**	0.2754	**0.0099**
Straightness	**0.0349**	0.3158	*0.0946*
Mean contraction	**0.0453**	*0.0892*	0.1451
Hausdorff dimension	*0.0545*	0.5955	0.1031
Mean fragmentation	**0.0101**	0.6274	**0.0132**

Values reflect *p* levels calculated using the Shapiro–Wilk normality test for each measure as indicated in each row, for each of the three data sets as indicated on the top of each column. Distributions that are unlikely to reflect a normal distribution are highlighted in italics (*p* < 0.1) and bold (*p* < 0.05)

**Table 4:** Minimal, median, mean, and maximal values of each morphological parameter distribution as indicated in each row, for each of the three data sets as indicated above the columns

Subjective class	Virus				Alexa				Biocytin			
	Min	Median	Mean	Max	Min	Median	Mean	Max	Min	Median	Mean	Max
Number of stems	3	6	6.03	11	2	4	4.63	8	3	7	6.45	9
Stem diameter—mean	0.68	2.16	2.17	3.22	1.17	1.75	1.97	3.88	1.67	2.5	2.49	3.95
Stem diameter—sum	6.11	12.7	12.55	18.93	4.6	8.6	8.94	15.5	10.2	15.13	16.98	32.6
Stem diameter—maximum	1.46	3.5	3.32	4.67	1.6	2.5	2.66	5.1	2.2	3.33	3.62	5.1
Stem directionality	0.12	0.3665	0.41	0.865	0.127	0.384	0.42	0.749	0.131	0.458	0.46	0.881
Dendrites—total length	1260	2311	2634.67	7006	372	1328	1423.93	3458	860	2974	3098.48	5764
Dendrite diameter—mean	0.81	2.045	2.05	3.45	1.1	2.5	2.46	4.1	1.53	2.83	2.98	5.87
Dendrites—longest single path length	170	330	367.69	906	92	268	295.56	523	257	436	468.52	1076
Number of bifurcations	6	16.5	20.22	43	3	8	9.11	17	3	12	12.48	31
Local bifurcation angle—mean	61.4	74.95	74.96	89.4	41.8	65.8	70.11	97.7	48	73	73.10	98.3
Remote bifurcation angle—mean	58.5	84.2	84.04	107.8	26.2	87.6	84.00	114.9	49.6	90.3	86.82	122.3
Number of branches	14	34.5	42.78	90	8	20	21.11	40	8	28	29.62	68
Branch order—maximum	3	6	6.81	13	2	4	4.67	9	3	4	4.72	9
Number of tips	1	10.5	11.94	33	0	7	8.04	19	3	12	12.66	31
Number of cut tips	0	7	6.08	11	0	4	3.59	9	1	3	3.76	13
Number of tips—total	9	16.5	18.03	38	5	11	11.63	23	6	16	16.41	35
Soma area	112	188	195.04	304	97	124	131.19	242	150	246	252.69	444
Hull volume (10^4^ µm^3^)	13.99	49.00	121.050	2062.270	6.930	49.24	60.25	160.13	23.53	115.07	192.64	1293.82
Soma-to-hull distance	0	11.621	14.55	51.332	0	6.218	6.64	24.628	0	7.258	10.54	37.675
Soma-to-center of gravity distance	8.445	30.697	37.67	133.654	8.912	30.76	52.69	201.59	7.417	51.762	59.69	131.578
Reach—maximum	65	121	139.53	265	35	154	145.56	298	95	165	183.62	420
Straightness	0.129	0.368	0.42	0.813	0.19	0.49	0.51	0.81	0.21	0.39	0.40	0.79
Mean contraction	0.59	0.695	0.71	0.83	0.53	0.68	0.69	0.79	0.5	0.6	0.61	0.8
Hausdorff dimension	1.05	1.18	1.20	1.47	1	1.1	1.11	1.28	1.06	1.14	1.16	1.3
Mean fragmentation	43.8	86.75	92.12	173.6	43.5	73.5	76.04	115	54	96.6	97.60	184.1

We then asked which of the objectively defined morphological parameters could best be used to describe the subjectively perceived range of variability from “curly” to “straight” by calculating the Spearman correlation between the subjectively assigned categories (curly, ambiguous and straight) and each of the measured parameters (Table 5, left side). Of all the different measures describing dendritic tree shape, straightness best corresponded to our subjective categorization across all three datasets; therefore, we chose this measure as an objective representation of a neuron’s position along the curly–straight continuum. Notably, besides measures directly aimed at describing the dendritic tree shape we found that in each data set at least one other measure was also correlated with the subjectively assigned classes (see Table 5); for example, in all three datasets the number of dendrite tips was significantly correlated with subjective class such that the “straight” neurons had the fewest tips. Similarly, these correlations could be found with the straightness-parameter instead of subjective class; for example, the number of stems and branches are strongly correlated both to subjective class and straightness in the viral- and Alexa-labeled datasets (see Table 5). Correlation statistics between straightness and all other morphometric parameters are reported on the right side in Table 5, and as examples, correlations of straightness to the number of stems, branches and total length are shown in Fig. 2f–h for the data obtained from viral-labeled morphologies; correlations between these parameters in the other two data sets follow the same trends and are shown in Supplementary Fig. 1.

**Table 5:** Correlations between morphometric measures and subjective classification (left) and straightness (right)

Measure names	Correlations to subjective classification						Correlations to straightness					
	Viral-labeled		Alexa-labeled		Biotin-labeled		Viral-labeled		Alexa-labeled		Biotin-labeled	
	Rho	*p*	Rho	*p*	Rho	*p*	Rho	*p*	Rho	*p*	Rho	*p*
Subjective class							− 0.71	< **0.0001**	− 0.83	< **0.0001**	− 0.50	**0.005**
Number of stems	0.31	0.067	0.51	**0.007**	0.36	0.055	− 0.41	**0.013**	− 0.60	**0.001**	− 0.12	0.536
Stem diameter—mean	− 0.30	0.080	− 0.35	0.075	− 0.32	0.089	0.58	**0.000**	0.42	**0.030**	0.15	0.428
Stem diameter—sum	− 0.08	0.661	0.23	0.254	− 0.35	0.067	0.14	0.411	− 0.10	0.608	0.31	0.104
Stem diameter—maximum	− 0.22	0.210	− 0.32	0.103	− 0.18	0.361	0.24	0.158	0.37	0.058	0.11	0.567
Stem directionality	0.37	**0.027**	0.00	0.996	0.11	0.563	− 0.11	0.538	− 0.09	0.664	− 0.13	0.492
Dendrites—total length	0.52	**0.001**	0.32	0.106	− 0.02	0.915	− 0.47	**0.004**	− 0.25	0.212	0.12	0.544
Dendrite diameter—mean	− 0.41	**0.014**	− 0.69	< **0.0001**	− 0.28	0.145	0.53	**0.001**	0.52	**0.005**	0.47	**0.011**
Dendrites—longest single path length	0.33	0.051	− 0.09	0.660	− 0.16	0.399	− 0.53	**0.001**	0.02	0.906	− 0.19	0.328
Number of bifurcations	0.60	< **0.0001**	0.50	**0.008**	0.33	0.085	− 0.69	< **0.0001**	− 0.31	0.112	− 0.25	0.192
Local bifurcation angle—mean	− 0.07	0.702	− 0.26	0.194	− 0.29	0.132	− 0.13	0.459	0.50	**0.008**	− 0.11	0.554
Remote bifurcation angle—mean	0.28	0.095	0.33	0.096	0.05	0.815	− 0.26	0.119	− 0.41	**0.034**	− 0.14	0.466
Number of branches	0.62	< **0.0001**	0.54	**0.004**	0.36	0.052	− 0.70	< **0.0001**	− 0.38	0.051	− 0.19	0.334
Branch order—maximum	0.51	**0.002**	0.19	0.345	0.10	0.592	− 0.74	< **0.0001**	0.01	0.972	− 0.43	**0.020**
Number of tips	0.73	< **0.0001**	0.69	< **0.0001**	0.48	**0.008**	− 0.67	< **0.0001**	− 0.54	**0.004**	− 0.42	**0.025**
Number of cut tips	− 0.40	**0.017**	− 0.30	0.125	− 0.22	0.254	0.40	**0.015**	0.21	0.298	0.39	**0.036**
Number of tips—total	0.65	< **0.0001**	0.68	< **0.0001**	0.32	0.091	− 0.63	< **0.0001**	− 0.54	**0.003**	− 0.26	0.173
Soma area	0.15	0.391	0.04	0.855	− 0.15	0.426	− 0.04	0.816	0.05	0.806	0.29	0.131
Hull volume	− 0.42	**0.010**	− 0.43	**0.024**	− 0.48	**0.008**	0.50	**0.002**	0.45	**0.017**	0.49	**0.007**
Soma-to-hull distance	− 0.02	0.910	0.24	0.225	− 0.07	0.713	− 0.12	0.504	− 0.23	0.249	0.09	0.649
Soma-to-center of gravity distance	− 0.47	**0.004**	− 0.63	< **0.0001**	− 0.41	**0.026**	0.48	**0.003**	0.67	< **0.0001**	0.40	**0.034**
Reach—maximum	− 0.54	**0.001**	− 0.72	< **0.0001**	− 0.52	**0.004**	0.71	< **0.0001**	0.80	< **0.0001**	0.68	< **0.0001**
Straightness	− 0.71	< **0.0001**	− 0.83	< **0.0001**	− 0.50	**0.005**						
Mean contraction	− 0.60	< **0.0001**	− 0.79	< **0.0001**	− 0.58	**0.001**	0.57	< **0.0001**	0.77	< **0.0001**	0.44	**0.016**
Hausdorff dimension	0.58	< **0.0001**	0.43	**0.026**	0.36	0.058	− 0.69	< **0.0001**	− 0.44	**0.021**	− 0.19	0.334
Mean fragmentation	0.05	0.778	0.57	**0.002**	− 0.01	0.974	− 0.12	0.491	− 0.56	**0.002**	− 0.08	0.691

Values reflect Spearman correlation statistics with each of the morphological parameters as indicated in each row, for each data set as indicated at the top of each column. Rho reflects the strength and direction of the correlation; *p* values highlighted in bold indicate measures that were significantly correlated with the subjective classification/straightness, respectively

**Fig. 3:**
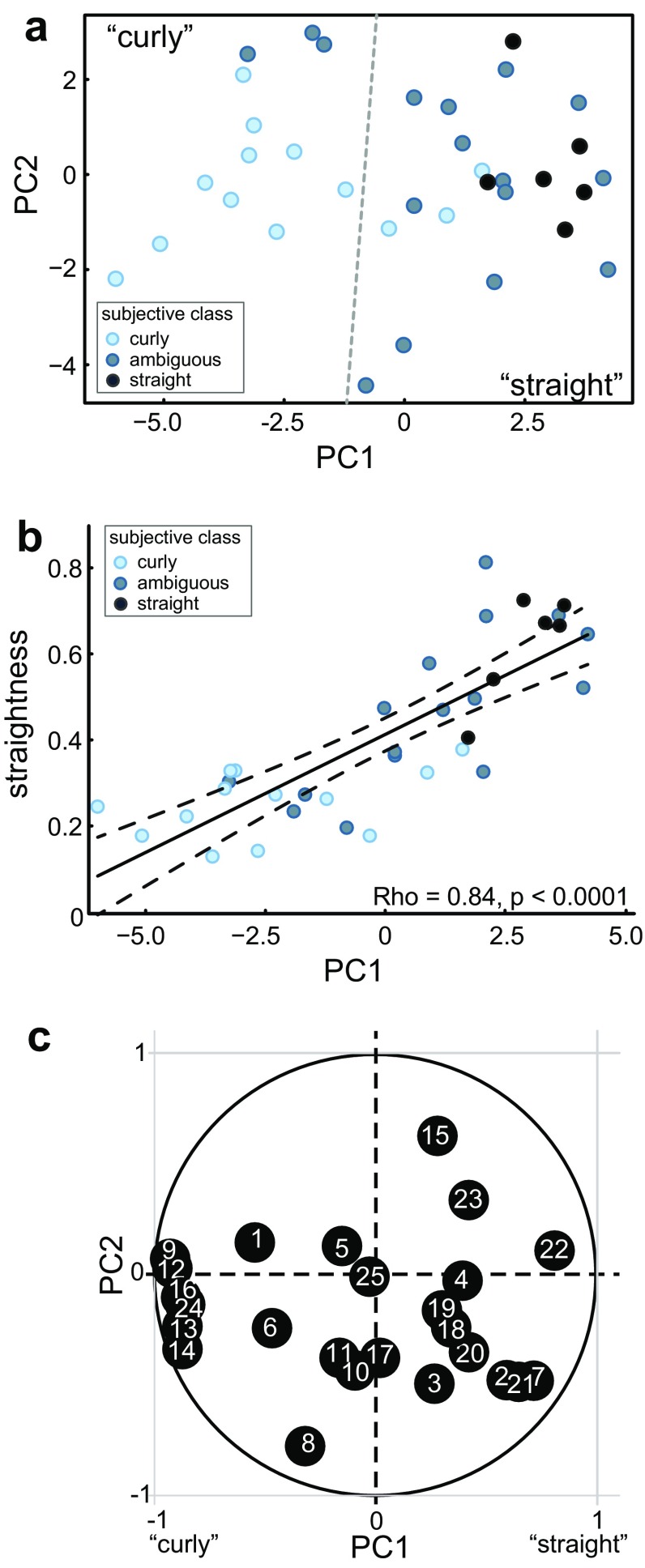
Algorithmic classification does not reveal clearly separated clusters. **a** Algorithmic classification shown as a scatter along the first two principal components (PCs) of separation for the viral-labeled data set. The grey dashed line marks the division into “curly” and “straight” groups as determined by a *K*-means algorithm; fill color represents subjective classification, as indicated. Note that the separation along the first principal component (PC1) appears to correspond to the subjective classification into morphological subtypes: subjectively “straight” neurons occupy the far-right side of the distribution while “ambiguous” and “curly” neurons are found in the middle and to the left. The slight mismatch between the subjective and algorithmic classification into “curly” and “straight” morphological types is another indication that seeking a quantitative justification for the subjective typification is futile. **b** Correlation of the main axis of separation to “straightness” in the viral-labeled data set; fill color represents subjective classification as indicated. **c** Relative contributions of the 25 morphometric parameters to the principal component separation in the viral-labeled data set. Numbers in circles correspond to the measures as listed in Table 1. The closer a parameter is to 1, the more it contributed to the separation in the PC space, in the direction indicated by its position within the unit circle; a parameter located at the origin did not contribute to the PC separation

Taking another approach to assessing which properties might best distinguish “curly” from “straight” morphologies we performed principal component analysis (PCA) and *K*-means clustering on the quantified morphological data (see “Methods”). If distinct morphological classes could be defined based on a combination of parameters, then dimensionality reduction of the data through PCA would result in a gap between groups of data points belonging to different classes. Due to the previously mentioned quantitative differences between the three data sets, the PCA-decomposition and *K*-means clustering results also vary quantitatively across data sets; nonetheless, the obtained results were qualitatively similar in each case, and are shown for viral-labeled data in Fig. 3 while the results of the same analyses performed on the patch-filled data sets are provided in Supplementary Fig. 2.

As shown in Fig. 3a, we found that a clear gap between groups of data points did not become apparent in the distribution of the data along the first two principal components (PC1 and PC2) of the PCA-decomposed morphometric data. To get an objective distinction into two groups despite this result, we applied a *K*-means clustering algorithm to the data as represented along the principal component axes (see “Methods”) and found that the algorithmic distinction between groups was defined almost exclusively along PC1; this is shown in Fig. 3a by the almost vertical grey dashed line marking the border between the two clusters. More than that, PC1 appeared to follow our subjective classification of the IO neuron morphological types; this is apparent in Fig. 3a in that most morphologies that were subjectively classified as being “curly” are found on the left side, while subjectively “straight” morphologies are all found on the right and “ambiguous” morphologies are mostly in between. Thus, it seems that PC1 closely follows the curly-to-straight continuum, and that “curly” and “straight” are indeed relevant descriptors of the morphological variability among IO neurons, i.e., a classification based on features unrelated to morphological “straightness” was not found. This idea was also reflected in the strong and significant correlation between PC1 and straightness (Fig. 3b, Rho = 0.84, *p* < 0.0001).

Our quantified morphometric data set contains multiple parameters aimed at describing the overall shape of dendritic trees; such parameters are correlated with each other by definition, and this may artificially cause the main principal component to follow measures of dendritic tree shape. However, as depicted in Fig. 3c where the relative contribution of each measure to the first two PCs is displayed on a scale from 0 to 1 for the viral-labeled morphologies, measures such as the number of stems and branches also contributed strongly to the separation along PC1. This shows that properties not directly describing dendritic tree shape also vary systematically with the measured straightness of the morphologies, and further strengthens our confidence that the curly–straight axis is the most relevant descriptor of morphological variability in the IO neuron population.

Taken together, the results described so far do not support the idea that IO neurons could or should be classified into subtypes based on their morphological appearance. Furthermore, these results indicate that a description of the morphological variability based on the simple straightness-measure is as informative as a description based on a decomposition of the quantified data.

### Non-isomorphic IO dendrite fields

**Fig. 4:**
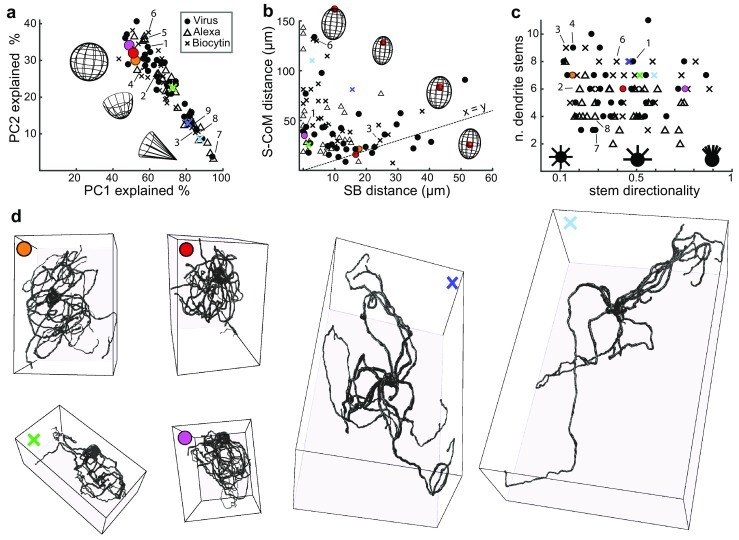
IO neuron morphologies with spherical dendritic fields and somata in the center are rare. **a** Scatter plot showing the percentage of variance explained by the first two principal components of the decomposition of IO neuron morphologies. The schematic line drawing insets in the plot illustrate the transition from “spherical” to “directional” dendritic field shapes. Colored points correspond to examples shown in panel **d**, while numbered points refer to examples shown in Fig. 1f. Symbols correspond to morphologies from the three datasets as indicated. **b** Scatter plot showing the distance from the soma to the extrapolated border of the neuron’s dendritic field (soma-border (SB) distance) relative to the distance from the soma to the center of mass of the dendritic arbor (S-CoM distance). Schematic line drawings illustrate the transition from “eccentric” to “centered” somata within an idealized, ovaloid dendritic field shape. Dotted line depicts unity, highlighting that the majority of neurons have somata much closer to the border than to the center of the volume they occupy. Numbers, symbols and colors used as in** a**.** c** Distribution of dendritic stem directionality with respect to number of stems. Insets in the plot schematically depict the variation from isomorphic (left) to directional (right). Note that the morphologies shown as examples in Figs. 1f and 4d have mostly isomorphically extending dendrite stems. Numbers, symbols and colors used as in** a**.** d** Additional examples of IO neuron morphologies. Colored circles denote morphologies from the viral-labeled data set; colored *x*’s denote morphologies from the biotin-labeled data. The orange and red morphologies are the only two examples in our library in which dendrites densely surround the soma on all sides. The morphologies marked with green and pink exemplify extreme (though not infrequent) examples of soma eccentricity. The morphologies marked with blue and cyan are examples of extremely extensive IO neuron morphologies with dendritic trees spreading far and wide in almost every direction around the soma. Note that the scale in the reconstructions varies according to viewing angle; somata are 15–18 µm in diameter

Non-isomorphic, or “pyriform” IO neuron dendritic fields have been described as early as the anatomical work of Ramón y Cajal (first published between 1905 and 1911); however, it has been assumed that such directionality arises only in the proximity of borders of the IO or its different subnuclei and that IO neurons residing within the main IO volume have roughly spherical shapes with somata surrounded by dendrites on all sides (Ramón y Cajal 1995; Scheibel and Scheibel 1955). Contrary to this description we found that neurons with directionally extended dendritic trees were also regularly encountered at distances far (> 75 µm) removed from boundaries of IO subnuclei (see Fig. 1a, g). In the following paragraphs we present two descriptors of dendritic directionality in IO neurons, one pertaining to the distribution of dendrites within the 3D volume occupied by the neuron (Fig. 4a), and one pertaining to the location of the soma within the dendritic volume (Fig. 4b). As no more correlation statistics will be presented, data acquired using different labeling methods are shown overlaid in the same panel, using different symbols to mark the different data sets as indicated.

There are two distinct ways in which the dendritic arrangement of an individual IO neuron can be non-homogeneous. First, the neuron’s dendrites are not distributed evenly within a spherical volume. We quantified this by performing PCA on the *x*-, *y*-, *z*-coordinates of the dendritic tree of each individual morphology. The relative proportions of variance explained along each of the three principal components (PCs) of a decomposed morphology represent the “stretchedness” of the dendritic tree along the axes of 3D space; if dendrites are distributed evenly within a spherical volume, each PC would explain 33% of the variance. Figure 4a shows that a large portion of neurons occupy a highly uneven volume with the first PC explaining more than 60% of their ‘variance in space’, whereas very few neurons are even roughly spherically shaped. As the examples shown in Fig. 4d illustrate, there is a continuum of dendritic tree shapes ranging from spherical (orange, red, and pink examples) to ellipsoid (green) to conical (blue) and even flat (cyan) morphologies. Notably, while the “straightest” morphologies were almost always highly elongated (see positions of examples 7–9 from Fig. 1 and the examples marked with blue and cyan in Fig. 4d), very “curly” morphologies also tended to have elongated shapes (see Fig. 1, example 3 and the example marked with green in Fig. 4d).

Second, IO neuron somata are usually not located in the center of mass (CoM) of the dendrites; instead, we found that in more than 90% of all neurons the shortest distance between the soma and the border of the volume they occupy (soma-border (SB) distance) is smaller than the soma-CoM distance (Fig. 4b). This means that IO neuron dendrites do not uniformly occupy the space around the soma, but instead extend into a preferred direction. In contrast to the directionality of the overall dendritic mass, the directionality in the positioning of dendrite stems on the soma is distributed randomly (Fig. 4c), so that directionality arises because dendrites take a sharp turn as they emerge from the soma and branch profusely only in the main direction.

Taken together, these results show that IO neuron dendritic trees are directional and indicate that this directionality is a relevant feature of the network’s architecture.

### Influence of dendrite directionality on network connectivity

**Fig. 5:**
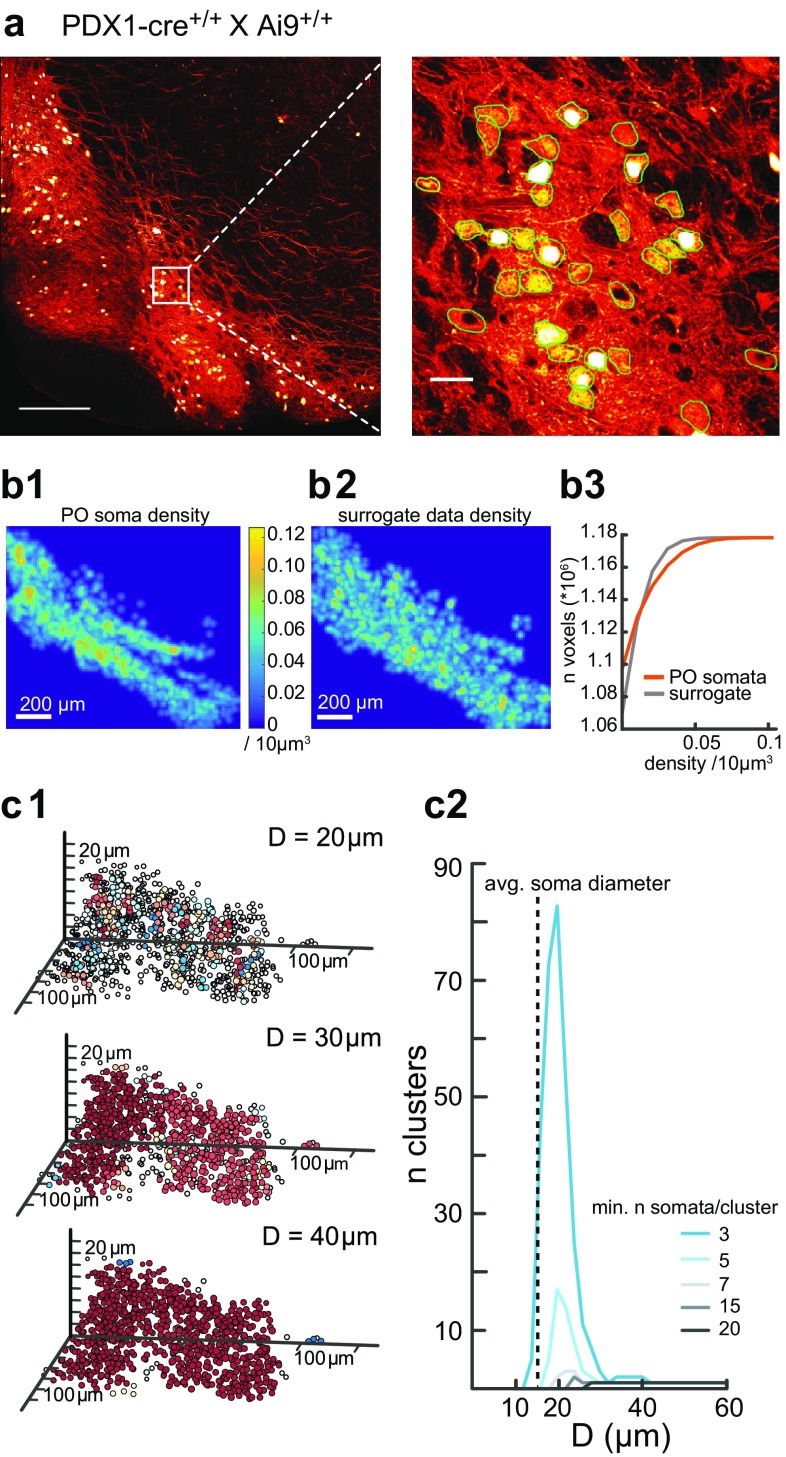
Anatomical clusters cannot be detected in the distribution of IO neuron somata. **a** Fluorescent labeling of all IO neuron somata. Left panel: coronal cross-section showing a full hemi-olive (scale bar 200 µm); right panel: magnification of the area delineated with a white square on the left (scale bar 25 µm). Detected somata are outlined in thin green lines; black holes are blood vessels. **b** Density distribution of somata. **b1** Soma density shown in a caudal view projection for the principal olive (PO). **b2** Same as **b1** but for shuffled surrogate data. **b3** Comparison of soma densities per 10µm^3^ voxel. Note that while the PO data has more high-density “hotspots” as well as “empty” regions (see “Methods”), density gradients are too weak to delineate anatomical clusters of somata. **c** Detection of clusters using the DBSCAN clustering algorithm (see “Methods”), in which cluster membership is defined as a group of points where each point is at most *D* µm removed from another point in the cluster. **c1** 3D-representation of clustering in the medial accessory olive (MAO) for different values of *D* as indicated (the minimal number of somata per cluster was set to 3). **c2** Total number of distinct clusters for different minimal cluster sizes as indicated by color-code. Dashed line represents average soma diameter. Note that multiple clusters are only detected at very short (< 20 µm) inter-soma distances, while the entire IO becomes a single cluster at inter-soma distance as short as 40 µm

As a final step in this anatomical investigation, we examined how the morphological variability and dendritic directionality might interplay in determining connectivity in the IO network. To this end, we first examined the distribution of IO neuron somata within the volume of the nucleus by manually reconstructing all 11,800 somata from one side of an entire rostro-caudal extent of an IO (Fig. 5a, “Methods”). While we found that the distribution of IO neuron somata is less homogeneous than would be expected if they were distributed uniformly within the IO volume (Fig. 5b), the inhomogeneities in the somata distribution were too weak to define anatomically segregated groups of neurons based on inter-soma distances alone. In fact, distance-based algorithmic clustering of somata showed that anatomically, somata are all grouped together into a single large cluster for inter-soma distances as small as 40 µm (Fig. 5c). Since all reconstructed morphologies have a reach of at least 35 µm, and the majority reach beyond 100 µm (see Fig. 2b), this result would indicate that IO neurons form a single, large interconnected mesh network. However, this assessment does not take into consideration that IO neuron dendritic trees can be strongly directional, as described in the previous paragraphs.

**Fig. 6:**
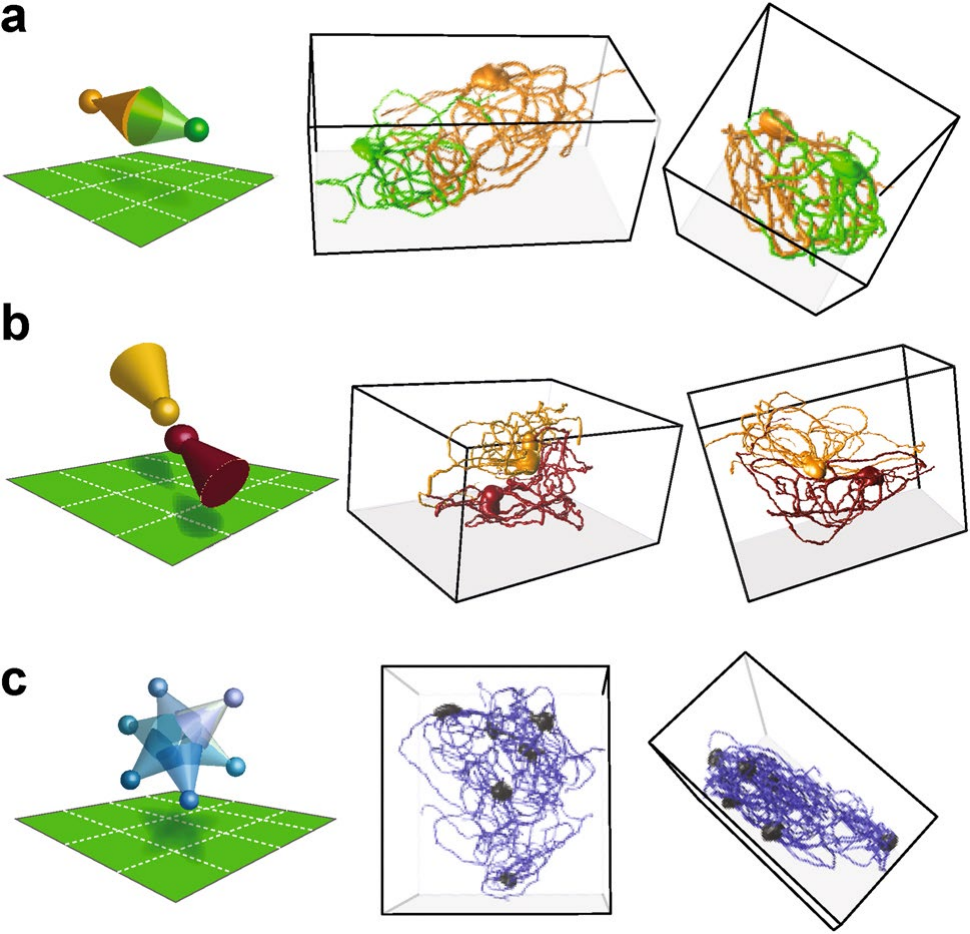
IO neuron dendritic tree arrangements relative to their neighbors suggest anatomical clustering of dendro-dendritic connectivity in the network. Schematic illustrations of dendritic field positioning are shown on the left, while the two right panels show reconstructed morphologies from two different viewing angles (**a–c**). **a** A pair of neurons with overlapping, directional dendritic trees. **b** A pair of neurons with proximally placed somata, but non-overlapping dendritic fields. **c** A group of neurons with somata residing at the outer rim of their overlapping dendritic fields

What connectivity properties may be bestowed on the IO network by the directionality in dendritic trees? We obtained data indicative of an answer to this question in experiments where labeling was less sparse than described so far, allowing us to occasionally visualize pairs or groups of neighboring neurons. In this material we observed that pairs of neighboring, directional morphologies were arranged such that their dendritic fields either expressly overlapped (Fig. 6a) or avoided each other nearly entirely (Fig. 6b). Furthermore, in rare cases where many nearby neurons could be reconstructed, their dendrites extensively overlapped and somata were located at the outer rim of the group of neurons (Fig. 6c). Thus, an attractive possibility is that the directionality of IO neuron dendritic trees, as well as their varying dendritic tree shapes, delineate anatomically segregated areas of mostly dense or more sparse connectivity.

## Discussion

In this study we provide a detailed, quantitative description of the morphological properties of a large sample of individual IO neurons and show that the heterogeneity in dendritic tree shapes spans a continuum between the “curly” and “straight” morphological types, defying this classical categorization. Furthermore, we find that IO neuron dendritic arbors are often clearly directional. In combination with our examination of their spatial distribution within the IO volume and their orientation relative to each other this leads to new ideas regarding the layout of connectivity within the IO network.

In the following paragraphs, we will first discuss some issues related to the description and classification of IO neuron morphologies, before delving into questions related to the significance of our findings for IO network architecture.

### Morphological characterization of IO neurons

Defining cell types has always been a major undertaking in neuroscience, as the layout of neuronal structures is of direct consequence to the connectivity, and thereby the function, of neuronal systems (Mukamel and Ngai 2018). Neurons in the IO network have classically been described as belonging either to the “curly” or “straight” subtype; however, this classification has always been subjective, and generalizable quantitative definitions of the classes are lacking. In this study, we give a detailed quantitative description of IO neuron morphological properties and find that the inter-individual variability is best described as encompassing a continuum along the curly-to-straight axis. To our knowledge, the included 90 morphologies form the most extensive collection of IO neuron reconstructions to date. Nonetheless, there are several issues pertaining to the labeling, sampling and statistical analysis of our library of IO neuron morphologies that need to be addressed.

First, it should be noted that the different methods for staining individual IO neurons lead to slightly differing data sets. Sparse viral transfection with fluorescent reporter proteins effectively reveals full individual neurons with minimal staining in the background; thus, even the most densely twisting, extremely “curly” morphologies could be reconstructed in fine detail. In contrast, reconstructions made of neurons patch-filled with either Alexa or biocytin may often underestimate the full extent of the dendritic arborizations, as incomplete penetration of the dye can leave parts of dendrites invisible. In our library, this is reflected in the overall lower number of branches in both patch-filled data sets, and the relatively short overall length of Alexa-filled morphologies (see Fig. 2b–e; Table 2). Furthermore, the different sample preparation methods used for viral-labeled and patch-filled cells may result in geometrical inconsistencies due to tissue shrinking and/or deformation during the experiment. Nevertheless, the general similarity of measurement distributions across the three datasets strongly suggests that even somewhat incomplete and deformed morphologies provide reliable information on the extent of a neuron’s “curliness”.

Another point requiring consideration is that we selected neuron morphologies for reconstruction based on the completeness of their being contained within the slice (see “Methods”); and since “curlier” neurons tend to occupy smaller volumes, they were less likely than their “straighter” counterparts to be excluded based on having multiple dendrites cut off at the slice surface. This issue is particularly prominent in the neurons labeled with biocytin which were often very close to the slice surface and selected for reconstruction only if their dendrites could be seen to extend down into the slice, while being much less apparent in the Alexa-labeled data because care was taken to patch neurons residing deeper (> 40 µm) in the slice. In addition, and in contrast to the more homogenous tissue sample set obtained from perfusion-fixed brains, the shape of post-fixed acute slices is affected by details of the in vitro experiment, making it difficult to ascertain uniform geometry especially in *z*-dimension.

Given the extensive morphological heterogeneity and the fact that the morphologies in our library were selected for inclusion based on the completeness of the reconstruction, it should be noted that our sample encompassing 90 morphologies does not necessarily reflect the distribution of morphological properties in the IO neuron population in an accurate and statistically representative manner. It is possible that overlapping, yet distinct morphological categories could be characterized in the full population encompassing more than 20,000 neurons in a single mouse IO (see Fig. 5). The reasons enumerated above also preclude us from making any claims about the relative proportions of “curlier” and “straighter” morphologies in the IO neuron population based on the samples included in our library. Nevertheless, as our investigation uncovered the same trend of continuity in morphological properties in each of three independently acquired data sets, we can confidently state that if a categorization of IO neuron types does exist, the type of an individual IO neuron cannot be deduced with certainty from its morphological properties alone.

### Significance of morphological variability and directionality for network architecture

The IO network is often implicitly considered as a homogeneously coupled mesh of neurons. However, such an organization would be computationally inefficient, and possibilities for delineating functional neuronal subgroups through modulation of GJ coupling between IO neurons have been examined through theoretical and experimental approaches alike (Benardo and Foster 1986; De Zeeuw et al. 1998; Tokuda et al. 2013; Pereda et al. 2013; Kazantsev et al. 2003; Blenkinsop and Lang 2006; Chaumont et al. 2013; de Zeeuw et al. 2011). For example, functional subgroups could be defined by inhibitory inputs shunting GJ currents between IO neurons, thereby effectively decoupling them (Llinas 1974; Lefler et al. 2014). The results presented in this paper are relevant to our understanding of the mechanisms generating synchronized activity in groups of IO neurons because they suggest that alongside the dynamic modulation of electrical coupling, the layout of coupling in the IO network is also defined in the variable density of dendro-dendritic overlap between neighboring IO neurons. Specifically, our results show that IO neuron morphologies have directional shapes (see Fig. 4a) and that somata are most often found at an eccentric location within the dendritic volume (see Fig. 4b). Importantly, such directionality occurs regardless of the distance between an IO neuron’s soma and the border of the subnucleus it resides in (see Fig. 1a, g). Thus, it is evident that the distribution of IO neuron somata (see Fig. 5) by itself is not directly indicative of the layout of functional connectivity between individual IO neurons.

Further evidence for a structured layout of electrical coupling in the IO network comes from examining the orientation of IO neuron dendritic trees relative to those of their neighbors. Examples where nearby directional neurons are labeled imply that IO neurons with closely situated somata need not necessarily form electrical connections (see Fig. 6b), and that dendritic directionality can delineate small subsets of IO neurons whose dendrites overlap with each other (see Fig. 6c). Thus, it is likely that the dendritic directionality delineates boundaries between groups of neurons, such that neurons residing within the same group are coupled to each other more tightly than to other neurons in the network. A network architecture like this has been previously proposed (Torben-Nielsen et al. 2012) as a mechanistic explanation for experimental observations of synchronized activity in groups of nearby IO neurons and propagating waves of oscillatory activity in slices (Leznik et al. 2002; Rekling et al. 2012; Kølvraa et al. 2014). Furthermore, experiments using tracer-diffusion as a measure of GJ-connectivity between IO neurons have shown that the extent and strength of coupling is heterogeneous and that coupled neurons usually reside within the dendritic field of the primary labeled neuron (Hoge et al. 2011), which is in line with the idea that there exist anatomical boundaries between groups of neurons in the IO network.

In the same way that dendritic directionality likely underlies functional clustering of IO neurons, dendritic curliness is likely to be the structural correlate of especially extensive dendro-dendritic coupling. Considering this, we propose that the straighter and less-directional neurons may function to provide weaker electrical coupling across different clusters in the network, effectively forming “bridges” between them. In this scenario, “cluster neurons” and “bridge neurons” form functionally distinct IO neuron subtypes whose morphological appearance may coarsely correspond to the “curly” and “straight” morphological subtypes. However, variability in the cluster sizes and in the strength and remoteness of bridge-connections results in considerable variability in “cluster” and “bridge” neuron shapes, giving rise to a continuum of morphological properties rather than clearly defined classes.

**Fig. 7:**
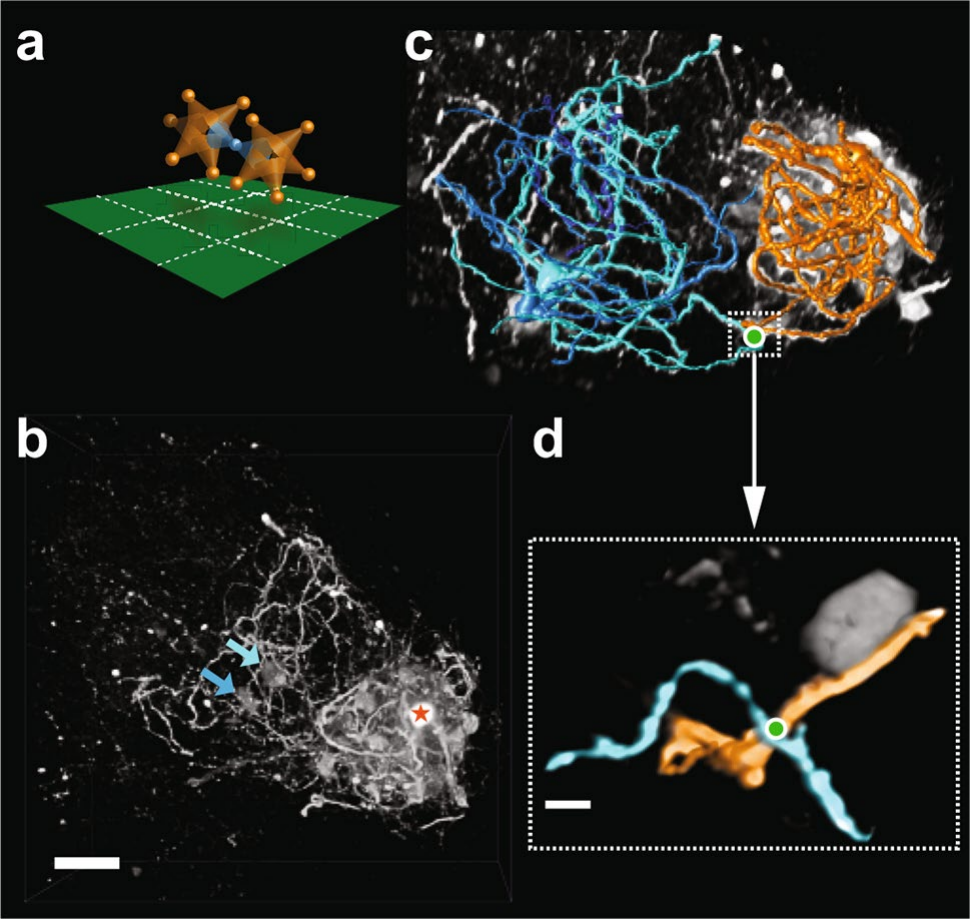
Example suggesting tight within-cluster coupling and weak inter-cluster coupling. **a** Schematic illustration of a “bridge neuron” (blue) providing weak coupling between two clusters (orange). **b** Confocal image stack *z*-projection showing a single directly labeled IO neuron (marked with an orange star) and a dense cluster of indirectly labeled neighbors, as well as two indirectly labeled neurons with somata residing outside the cluster (marked with blue and cyan arrows). Scale bar 50 µm. **c** Reconstructions of the neurons marked in **b**, revealing a point of close proximity between their dendrites. **d** High-magnification confocal *z*-stack image showing the area marked with a white box in **c**. Green dot marks a putative GJ-connection between the primary labeled neuron (orange-colored dendrite) and a “bridge neuron” (blue-colored dendrite). Scale bar 5 µm

A tantalizing example in line with such “cluster-bridge connectivity” is shown in Fig. 7. In this sample, a single patched neuron (Fig. 7b, reconstructed in orange) is accompanied by a number of densely overlapping dendritic arbors forming a compact cluster of neurons in a volume spanning the extent of the primary neurons’ dendritic field. Additionally, two neurons located further away from the primary labeled cell (indicated by blue arrows in Fig. 7c) were also labeled and could be resolved well enough to be partially reconstructed (blue and cyan reconstructions in Fig. 7d), revealing a location where a dendrite passes close by that of the directly labeled neuron (marked with a green dot in Fig. 7c, d). This raises the possibility of GJ-mediated coupling between the dense cluster and the “bridge neurons”.

In summary, our anatomical investigation of IO neurons showed that a binary classification into the classically described “curly” and “straight” morphological types is not justified as morphological heterogeneity is better described as varying along a continuous straightness-axis. In addition, the prevalence of directional over isomorphic dendritic fields implies that connectivity in the IO network is structured to support functional clustering. We propose that borders between anatomical clusters are delineated in the dense electrical coupling within groups of “cluster neurons”, and that coupling across such clusters is mediated by dedicated “bridge neurons”. The specific morphology of individual neurons forming clusters and bridges can both vary considerably, resulting in an apparent continuum of morphological properties.

However, the density of IO neuropil and the limitations of the present random-sampling approach preclude strong conclusions to be drawn from anatomical evidence alone, and further electrophysiological and imaging experiments detailing the relationship between the structure and activity of IO neurons will be required to confirm and refine any hypothesis about the hard-wired connectivity of the IO network.

## Electronic supplementary material

Below is the link to the electronic supplementary material.

Supplementary material 1 Supplementary Figure 1 (supplement to Figure 2) Morphological parameter correlations to straightness in the patch-labeled data sets. Distributions of number of dendrite stems (top), number of branch points (middle) and total dendritic length (bottom) with respect to straightness in the Alexa-labeled data (left column) and the Biocytin-labeled data (right column); the same distributions in the viral-labeled data set are displayed in Fig. 2f–h. Reported correlation statistics represent the strength and direction (Rho) and significance level (*p*) calculated using Spearman’s rank correlation test (see “Methods”). Correlations between straightness and all other morphological measures are reported in the right half of Table 2 for each of the three data sets (EPS 754 KB)

Supplementary material 2 Supplementary Figure 2 (supplement to Figure 3) Algorithmic classification does not reveal clearly separated clusters in two data sets of patch-filled morphologies. **a** Algorithmic classification shown as a scatter along the first two principal components (PCs) of separation for the Alexa-labeled (left) and Biocytin-labeled (right) data sets. The grey dashed line marks the algorithmically forced *K*-means grouping into “curly” and “straight”; fill color represents subjective classification, as indicated. In the Alexa-labeled data, as in the viral-labeled data, the separation into distinct groups (as determined by the *K*-means algorithm) spreads along PC1, whereas in the Biocytin-labeled data, the distinction is better represented along PC2. **b** Correlation between “straightness” and the first (top panel) and second (bottom panel) principal component of separation for the Alexa-labeled (left) and Biocytin-labeled (right) data sets. In the Alexa-labeled data the first two principal components of separation are both correlated with straightness. In contrast, only PC2 is significantly correlated with straightness in the Biocytin-labeled data. Nonetheless, the classification results are qualitatively similar in all three data sets in that the grouping assigned by the *K*-means algorithm draws a distinguishing line almost perpendicular to the principal component most strongly correlated to straightness. **c** Relative contributions of the 25 morphometric parameters to the principal component separation in the Alexa-labeled (left) and Biocytin-labeled (right) data sets. Numbers in circles correspond to the measures as listed in Table 1. The closer a parameter is to 1, the more it contributed to the separation in the PC space, in the direction indicated by its position within the unit circle; a parameter located at the origin did not contribute to the PC separation. Notably, the distribution of parameters contributing strongly to the separation along PC2 in the Biocytin-labeled data set is similar to the distribution of parameters contributing strongly to PC1 in the Alexa-labeled and viral-labeled data (see Fig. 3c) (EPS 1352 KB)
